# Response to Wiart, C. Lee *et al*., Inhibitory Effect and Mechanism of Antiproliferation of Isoatriplicolide Tiglate (PCAC) from *Paulownia coreana*. *Molecules* 2012, *17*, 5945-5951: A Note Regarding *Paulownia coreana*. Molecules 2013, 18, 2587-2588

**DOI:** 10.3390/molecules18033041

**Published:** 2013-03-07

**Authors:** Myeong-Sok Lee

**Affiliations:** Division of Biological Science and Research Center for Women’s Diseases, Sookmyung Women’s University, Seoul 140-742, Korea; E-Mail: mslee@sookmyung.ac.kr

In a Comment recently published in Molecules [1], Prof. C. Wiart took issue with our identification of the plant species used in our work as *Paulownia coreana*. Although this name was assigned by H. Uyeki in 1925 [2], appears as such in handbooks of Korean flora [3], and examples of its use can be found in the recent literature [4,5,6,7,8,9], after reviewing the arguments and references presented in [1], we now recognize that this is no longer considered an accepted species name, and therefore we wish to revise our assignment to *Pauwlonia tormentosa* (Thunb.) Steud. We thank Prof. Wiart for bringing this fact to our attention and apologize to the readership of *Molecules* for any confusion caused by our previous classification of the species.

Myeong-Sok Lee

## References

[1] Wiart C. (2013). Lee *et al*., Inhibitory Effect and Mechanism of Antiproliferation of Isoatriplicolide Tiglate (PCAC) from *Paulownia coreana*. *Molecules* 2012, *17*, 5945-5951: A Note Regarding *Paulownia coreana*. Molecules.

[2] Uyeki H. (1925). Bull. Agric. Forest Coll. Suigen (Suwon).

[3] Lee W.T. (1996). Lineamenta Florae Koreae.

[4] Kim H.Y., Oh Jong H. (1999). Screening of Korean Forest Plants for Rat Lens Aldose Reductase Inhibition (as material). Biosci. Biotechnol. Biochem..

[5] Han M.S., Noh E.W., Yun J.K. (2001). Differentiation of Phytoplasmas Infecting *Zizyphus jujuba* and *Paulownia coreana* Using PCR-RFLP. Plant Pathol. J..

[6] Kim J.K., Si C.L., Bae Y.S. (2007). Epimeric phenylpropanoid glycosides from inner bark of *Paulownia coreana* Uyeki. Holzforschung.

[7] Kim J.K., Si C.L., Bae Y.S. (2008). Phenylpropanoid glycosides from the leaves of *Paulownia coreana*. Nat. Prod. Res..

[8] Si C.L., Bae Y.S., Ni Y.H., Li S.M. (2010). Study on the Secondary Metabolites of *Paulownia coreana*. Planta Med..

[9] Kim J.K., Lee Y.S., Kim S.H., Bae Y.S., Lim S.S. (2011). Inhibition of Aldose Reductase by henylethanoid Glycoside Isolated from the Seeds of *Paulownia coreana*. Biol. Pharm. Bull..

